# Survival analysis and development of a prognostic nomogram for patients with malignant mesothelioma in different anatomic sites

**DOI:** 10.3389/fonc.2022.950371

**Published:** 2022-11-10

**Authors:** Shengteng Shao, Lei Sun, Kun Qin, Xiangfeng Jin, Tengfei Yi, Yuhong Liu, Yuanyong Wang

**Affiliations:** ^1^ Department of Thoracic Surgery, The Affiliated Hospital of Qingdao University, Qingdao, Shandong, China; ^2^ Department of Radiology, The Affiliated Hospital of Qingdao University, Qingdao, Shandong, China; ^3^ Department of Emergency, The Affiliated Hospital of Qingdao University, Qingdao, Shandong, China; ^4^ Department of Thoracic Surgery, Tangdu Hospital, Air Force Medical University, Xi’an, Shanxi, China

**Keywords:** malignant mesothelioma, survival, nomogram, different anatomic sites, SEER database

## Abstract

**Background:**

Malignant mesothelioma (MMe) is a rare and fatal cancer with a poor prognosis. Our study aimed to compare the overall survival (OS) of MMe patients across various sites and develop a prognostic model to provide a foundation for individualized management of MMe patients.

**Methods:**

From the Surveillance, Epidemiology, and End Results (SEER) database, 1,772 individuals with malignant mesothelioma (MMe) were identified. The X-tile software was used to identify the optimal cut-off point for continuous variables. The Kaplan–Meier method was employed to compare the survival of MMe across different sites. The Cox proportional hazards model was applied to identify the independent risk factors of overall survival (OS) and a nomogram was constructed.

**Results:**

In the survival analysis, MMe originating from the reproductive organs and hollow organs showed a relatively better prognosis than those originating from soft tissue, solid organs, and pleura. Age, gender, location, histological type, grade of differentiation, extent of disease, lymph node status, lymph node ratio (LNR), and chemotherapy were all found to be independent risk variables for the prognosis of MMe patients (P<0.05) in a multivariate Cox analysis and were included in the construction of nomogram. In the training and testing sets, the C-index of the nomogram was 0.701 and 0.665, respectively, and the area under the ROC curve (AUROC) of the 1-, 3-, and 5-year overall survival rate was 0.749, 0.797, 0.833 and 0.730, 0.800, 0.832, respectively. The calibration curve shows that the nomogram is well-calibrated.

**Conclusions:**

This is the first research to examine the prognosis of MMe patients based on the location. However, previous studies often focused on malignant pleural mesothelioma or malignant peritoneal mesothelioma with high incidence. Furthermore, a nomograph with good prediction efficiency was established according to the variables that influence patient survival outcomes, which provides us with a reference for clinical decision-making.

## Introduction

Malignant mesothelioma (MMe) is an uncommon and aggressive cancer (1) mostly associated with asbestos exposure (2, 3). Other causes of mesothelioma include exposure to other types of mineral fibers, radiation, chronic pleural inflammation, or germline and somatic inactivation of the BRCA1-associated protein 1 gene (BAP1) (4, 5). Most cases originate from the pleura, with a lower prevalence at other anatomical sites such as the peritoneum, genital organs, soft tissue, and other organs of the body (6).

Previous studies mostly focused on malignant mesothelioma originating in the pleura or peritoneum; and age, gender, histology, differentiation, lymph node status, chemotherapy, and other characteristics have been demonstrated to be independent prognostic factors (7–11).

It is worth mentioning that the overall concordance between biopsies and surgical resection specimens is 80.6% (12). Clinical N0 stage (cN0) malignant pleural mesothelioma (MPM) patients with lymph node metastasis have a poor prognosis (13). In addition, Leuzzi et al. found that the pathological N stage (pN), rather than the clinical N stage (cN), was the potential predictive factor affecting the overall survival (OS) (14). To avoid this error, all candidates enrolled in our research were diagnosed by positive histology recorded in the SEER database.

The prediction model of MMe has been reported in several population-based studies. Some studies have established a prognosis model of malignant pleural mesothelioma after surgical treatment or systemic chemotherapy (7, 15), and others have proposed a diagnostic model for early recognition of MM through machine learning technology (16). However, these analyses have often been confined to the pleura, peritoneum, or other specific sites and no comparison in prognosis has been drawn across the various anatomic sites. In our study, the survival outcomes of MMe patients from all sites documented in the SEER database were analyzed and the cohort from the SEER database was utilized to develop a novel nomogram to predict the overall survival (OS), thus providing a basis for the individualized management of MMe in various anatomic sites.

## Methods

### Patient collection and definition

The SEER 18 registries (2000–2018), which encompass roughly 28% of the population of the United States, were used to compile the data. ICD-O-3/WHO 2008 morphology codes 9050–9053 were used to identify patients with mesothelioma. And tumor behavior records that were malignant were included in the analysis. After initial screening, candidates were enrolled in the study.

The criteria of inclusion were as follows: (a) patients diagnosed with malignant mesothelioma according to ICD-O-3/WHO 2008 morphology codes; (b) patients diagnosed between 2004 and 2015; (c) Patients who have MMe as their first primary malignancy. The exclusion criteria were as follows: (a) Patients whose location was not otherwise specified (NOS); (b) Patients who did not have surgery and were just subjected to a biopsy or whose surgical status was unknown; (c) Patients without survival information; (d) The number of lymph nodes examined or the number of positive lymph nodes is unknown. Accordingly, a total of 1772 patients were included in our study. After strict inclusion and exclusion, the research object of this article is the primary malignant mesothelioma diagnosed pathologically from 2004 to 2015, which avoids the impact of secondary malignant tumors on the results. Moreover, the pathological information of lymph nodes in all subjects is complete, which can be further used to analyze the relationship between them and the prognosis of patients with malignant mesothelioma.

The data from the SEER database is free and publicly available; no institutional review board permission was required. The relevant clinical data were extracted, including the year of diagnosis, age, gender, marital status, race, location, histologic type, grade, extent of disease, TMN stage, T classification, lymph node status, regional nodes examined, regional nodes positive, distant metastasis, treatment (including surgery, radiotherapy, and chemotherapy), survival time and vital status.

The variables of age, number of lymph nodes examined and number of positive lymph nodes were extracted from the Seer database. The lymph node ratio (LNR) is the ratio of positive lymph nodes to examined lymph nodes. The optimal cut-off value was determined by X-tile software and the variables were subdivided into 3 groups to facilitate further statistical analysis. The specific group information was as follows: the age of the patients (<65, 65 -75 and > 75), number of nodules dissected (0, 1 - 11, 12 - 81), number of positive nodules (0, 1 - 3, 4 - 31, No dissection) and LNR (<0.003, 0.003 - 0.45, 0.4 5- 1, No dissection). Following the screening, the locations were sorted into the following seven groups: (a) Pleura; (b) Peritoneum, retroperitoneum; (c) Male genital organs, including testis and other male genital organs; (d) Female genital organs, including ovary and fallopian tube, round ligaments and uterine adnexa; (e) Soft tissues; (f) Solid organs, including lung, heart or mediastinum, thymus, liver, and spleen;(g) Hollow organs, including colon, small intestine, and stomach. The Seer surgery code was used to gather surgical information, which was divided into three types of procedures: palliative, radical, and not otherwise specified (NOS). Regional lymph nodes dissection was grouped into Yes and No/Unknown according to the records from the SEER database. Chemotherapy was considered to have been administered if the chemotherapy record stated Yes. According to the radiotherapy records in the Seer database, the remaining patients were considered to have received radiotherapy, excluding those who refused radiotherapy or whose radiotherapy status was unclear.

### Statistical analysis and nomogram development

The optimal cut-off points of the continuous variables, including age, the number of lymph nodes dissected, the number of positive lymph nodes, and LNR were identified by the X-tile program, which utilized the minimum P-value method to find them. The specific group information is displayed in **Table 1**. Survival analysis was accomplished by the Kaplan–Meier approach and the log-rank test. The enrolled patients were divided into the training and testing groups based on their age at diagnosis. Significant variables in the univariate Cox proportional hazards regression model were entered into a multivariate analysis using LR stepwise selection with the Akaike information criterion (AIC). The predictor coefficients were then calculated using data from 1,332 patients diagnosed between 2007 and 2015. The researchers calculated the hazard ratios (HRs) and 95 percent confidence intervals (CIs). Significant covariates in the stepwise Cox model were used to construct the nomogram. Using data from 440 patients diagnosed between 2004 and 2006, the discrimination and calibration of the model were then validated.

**Table 1:** The demographic, tumor, and treatment characteristics for malignant mesothelioma (MMe) patients in training and validation cohorts.

Variable	Training set	Testing set	Overall
	(N = 1332)	(N = 440)	(N = 1772)
**Marital status**			
Married	869 (65.2%)	311 (70.7%)	1180 (66.6%)
Divorced/Widowed/Separated	241 (18.1%)	76 (17.3%)	317 (17.9%)
Unmarried	176 (13.2%)	48 (10.9%)	224 (12.6%)
Unknow	46 (3.5%)	5 (1.1%)	51 (2.9%)
**Race**			
White	1206 (90.5%)	409 (93.0%)	1615 (91.1%)
Black	76 (5.7%)	18 (4.1%)	94 (5.3%)
Others	50 (3.8%)	13 (3.0%)	63 (3.6%)
**Age, year**			
<65	562 (42.2%)	194 (44.1%)	756 (42.7%)
65 - 75	475 (35.7%)	156 (35.5%)	631 (35.6%)
> 75	295 (22.1%)	90 (20.5%)	385 (21.7%)
**Sex**			
Male	939 (70.5%)	320 (72.7%)	1259 (71.0%)
Female	393 (29.5%)	120 (27.3%)	513 (29.0%)
**Location**			
Pleura	990 (74.3%)	332 (75.5%)	1322 (74.6%)
Peritoneum/Retroperitoneum	260 (19.5%)	76 (17.3%)	336 (19.0%)
Hollow organ	6 (0.5%)	2 (0.5%)	8 (0.5%)
Solid organ	23 (1.7%)	7 (1.6%)	30 (1.7%)
Male genital organ	38 (2.9%)	16 (3.6%)	54 (3.0%)
Female genital organ	8 (0.6%)	3 (0.7%)	11 (0.6%)
Soft tissue	7 (0.5%)	4 (0.9%)	11 (0.6%)
**Histologic type**			
Fibrous	100 (7.5%)	46 (10.5%)	146 (8.2%)
Epithelioid	664 (49.8%)	172 (39.1%)	836 (47.2%)
Biphasic	166 (12.5%)	37 (8.4%)	203 (11.5%)
Unknown	402 (30.2%)	185 (42.0%)	587 (33.1%)
**Differentiation**			
Well	64 (4.8%)	33 (7.5%)	97 (5.5%)
Moderately	15 (1.1%)	3 (0.7%)	18 (1.0%)
Poorly/Undifferentiated	110 (8.3%)	42 (9.5%)	152 (8.6%)
Unknown	1143 (85.8%)	362 (82.3%)	1505 (84.9%)
**Extent of disease**			
Localized	166 (12.5%)	50 (11.4%)	216 (12.2%)
Regional	288 (21.6%)	98 (22.3%)	386 (21.8%)
Distant	847 (63.6%)	286 (65.0%)	1133 (63.9%)
Unknown	31 (2.3%)	6 (1.4%)	37 (2.1%)
**Stage**			
I	195 (14.6%)	50 (11.4%)	245 (13.8%)
II	155 (11.6%)	62 (14.1%)	217 (12.2%)
III	350 (26.3%)	80 (18.2%)	430 (24.3%)
IV	399 (30.0%)	105 (23.9%)	504 (28.4%)
Unknown	233 (17.5%)	143 (32.5%)	376 (21.2%)
**T**			
T1	237 (17.8%)	73 (16.6%)	310 (17.5%)
T2	281 (21.1%)	88 (20.0%)	369 (20.8%)
T3	322 (24.2%)	76 (17.3%)	398 (22.5%)
T4	238 (17.9%)	85 (19.3%)	323 (18.2%)
Unknown	254 (19.1%)	118 (26.8%)	372 (21.0%)
**N**			
N0	756 (56.8%)	222 (50.5%)	978 (55.2%)
N1/N2/N3	321 (24.1%)	72 (16.4%)	393 (22.2%)
Unknown	255 (19.1%)	146 (33.2%)	401 (22.6%)
**M**			
M0	942 (70.7%)	269 (61.1%)	1211 (68.3%)
M1	210 (15.8%)	33 (7.5%)	243 (13.7%)
Unknown	180 (13.5%)	138 (31.4%)	318 (17.9%)
**Surgical type**			
Palliative	978 (73.4%)	309 (70.2%)	1287 (72.6%)
Radical	300 (22.5%)	110 (25.0%)	410 (23.1%)
NOS	54 (4.1%)	21 (4.8%)	75 (4.2%)
**Lymph nodes dissection**			
Yes	438 (32.9%)	121 (27.5%)	559 (31.5%)
No/Unknown	894 (67.1%)	319 (72.5%)	1213 (68.5%)
**Number of nodules dissected**			
0	892 (67.0%)	315 (71.6%)	1207 (68.1%)
1 - 11	272 (20.4%)	84 (19.1%)	356 (20.1%)
12- 81	168 (12.6%)	41 (9.3%)	209 (11.8%)
**Number of positive nodules**			
0	247 (18.5%)	71 (16.1%)	318 (17.9%)
1 - 3	111 (8.3%)	32 (7.3%)	143 (8.1%)
4 - 31	82 (6.2%)	22 (5.0%)	104 (5.9%)
No dissection	892 (67.0%)	315 (71.6%)	1207 (68.1%)
**LNR**			
<0.003	248 (18.6%)	71 (16.1%)	319 (18.0%)
0.003 - 0.45	101 (7.6%)	23 (5.2%)	124 (7.0%)
0.45 - 1	91 (6.8%)	31 (7.0%)	122 (6.9%)
No dissection	892 (67.0%)	315 (71.6%)	1207 (68.1%)
**Radiation**			
Yes	225 (16.9%)	80 (18.2%)	305 (17.2%)
No/Unknown	1107 (83.1%)	360 (81.8%)	1467 (82.8%)
**Chemotherapy**			
Yes	806 (60.5%)	231 (52.5%)	1037 (58.5%)
No/Unknown	526 (39.5%)	209 (47.5%)	735 (41.5%)

For statistical analyses, we utilized R software version 4.1.2. The nomogram was created and drawn using the regplot package. A two-sided P value of less than 0.05 was considered statistically significant.

## Results

### Population characteristics

A total of 1,772 patients from the National Cancer Institute’s SEER program were enrolled in this research. The patients diagnosed between 2007 and 2015 were assigned to the validation group and those diagnosed between 2004 and 2006 to the training group. The demographic, pathology and treatment information for the training (n = 1,332) and testing cohorts (n = 440) are displayed in **Table 1**.

The majority of patients in the primary cohort were married (66.6%), white (91.1%), aged <75 (78.3%), male (71.0%), and had MMe originating from the pleura (74.6%). Epithelioid histology was seen in 836 individuals (47.2%), biphasic histology in 203 patients (11.5%), and fibrous histology in 146 patients (8.2%), but the degree of differentiation in the majority of MMe patients was unknown (84.9%).

There were 245 patients with stage I (13.8%), 217 with stage II (12.2%), 430 with stage III (24.3%), 504 with stage IV (28.4%), and 376 with an unknown stage (21.2%). Distribution of the T stage was 310 (17.5%), 369 (20.8%), 398 (22.5%), 323 (18.2%) and 372 (21.0%) for T1, T2, T3, T4 and unknown, respectively. 393 (22.2%) patients had lymph node metastasis, 242 (13.7%) with distant metastasis.

Most patients (72.6%) underwent palliative surgery, and 410 patients (23.1%) underwent radical surgery. Most patients (68.5%) did not undergo lymph node dissection. Only 305 patients (17.2%) were treated with radiotherapy. Chemotherapy was performed on 1,037 patients (58.5%).

### Subgroup analysis of different anastomotic sites

**Figure 1:**
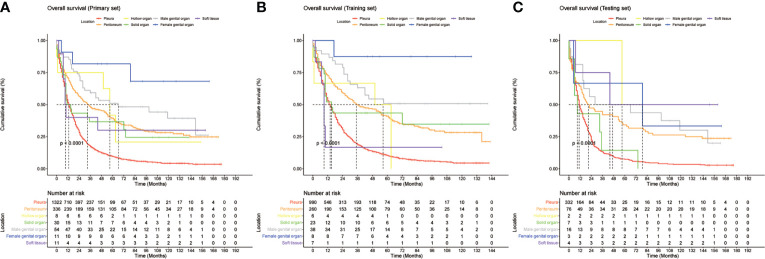
Kaplan-Meier curves of overall survival (OS) based on different primary sites in **(A)**, entire cohort, **(B)**, training cohort, and **(C)**, validation cohort.

**Figure 2:**
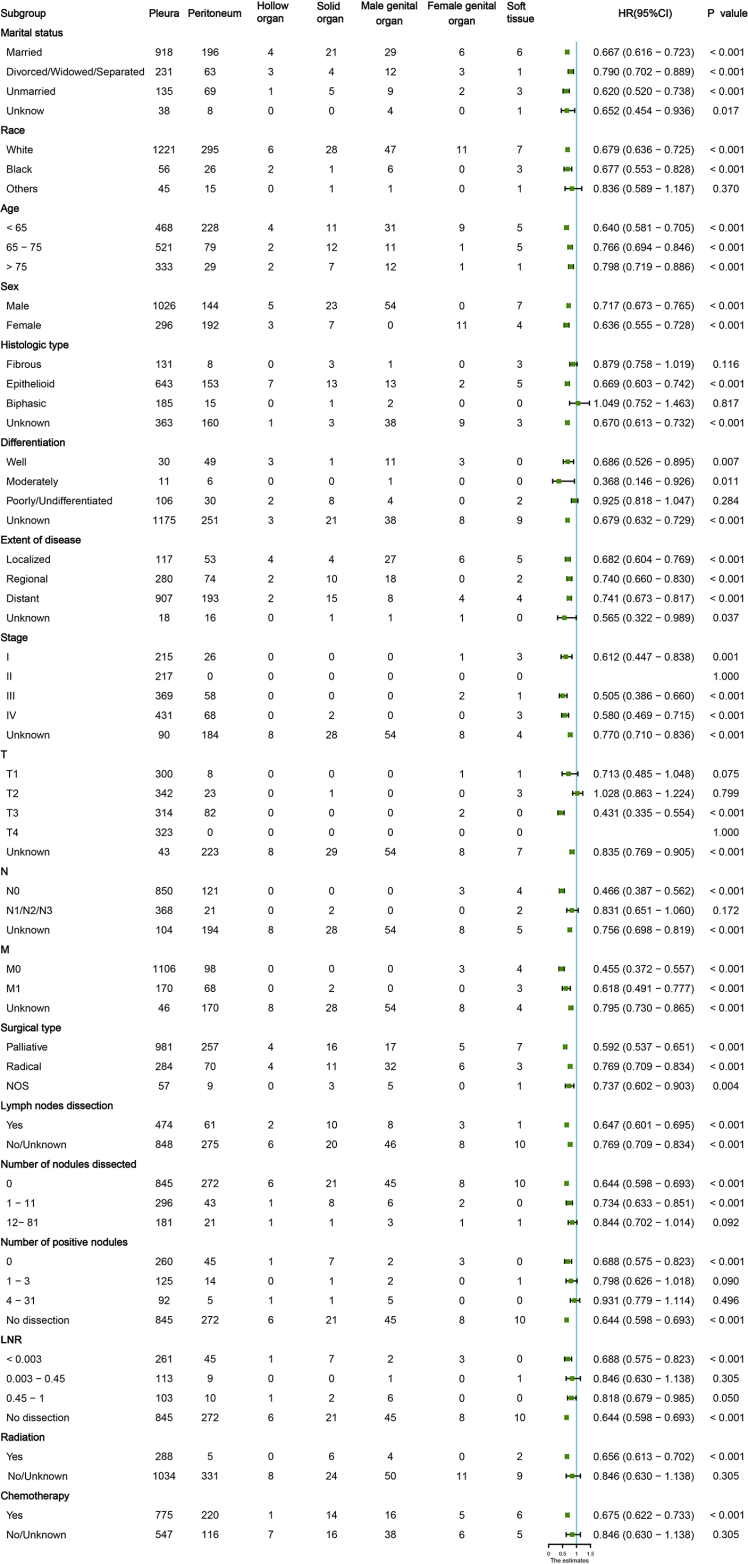
Subgroup analysis of the baseline characteristics in patients with malignant mesothelioma (MM) at different sites.

The MMe death rate reached 100% during the follow-up period in the two cohorts. In the whole group, the median OS was 16 months (95% CI 14.811–17.189 months). MMe originating from soft tissue, solid organs and pleura had a poor prognosis, whereas MMe originating in reproductive organs and hollow organs showed a relatively better prognosis. The specific median survival times and their 95% CIs for different anatomical sites are shown in **Table 2**. The survival curve trends of the training and testing cohorts were similar to the full set (**Figure 1**). Notably, the subgroup analysis revealed statistically significant differences in the prognostic variables influencing survival time across different sites of malignant mesothelioma (**Figure 2**).

**Table 2:** Median overall survival (OS) and 95%CI of all malignant mesothelioma (MMe) patients and MMe patients in different sites.

Location			95%CI	
	Median OS (month)	SE	lower.95	upper.95
Pleura	13	0.532	11.957	14.043
Peritoneum	33	5.986	21.267	44.733
Hollow organ	57	6.882	43.511	70.489
Solid organ	11	2.739	5.632	16.368
Male genital organ	66	36.394	0.000	137.333
Female genital organ	–	–	–	–
Soft tissue	9	1.581	5.901	12.099
Overall	16	0.607	14.811	17.189

### Screening for predictive factors and nomogram construction

**Figure 3:**
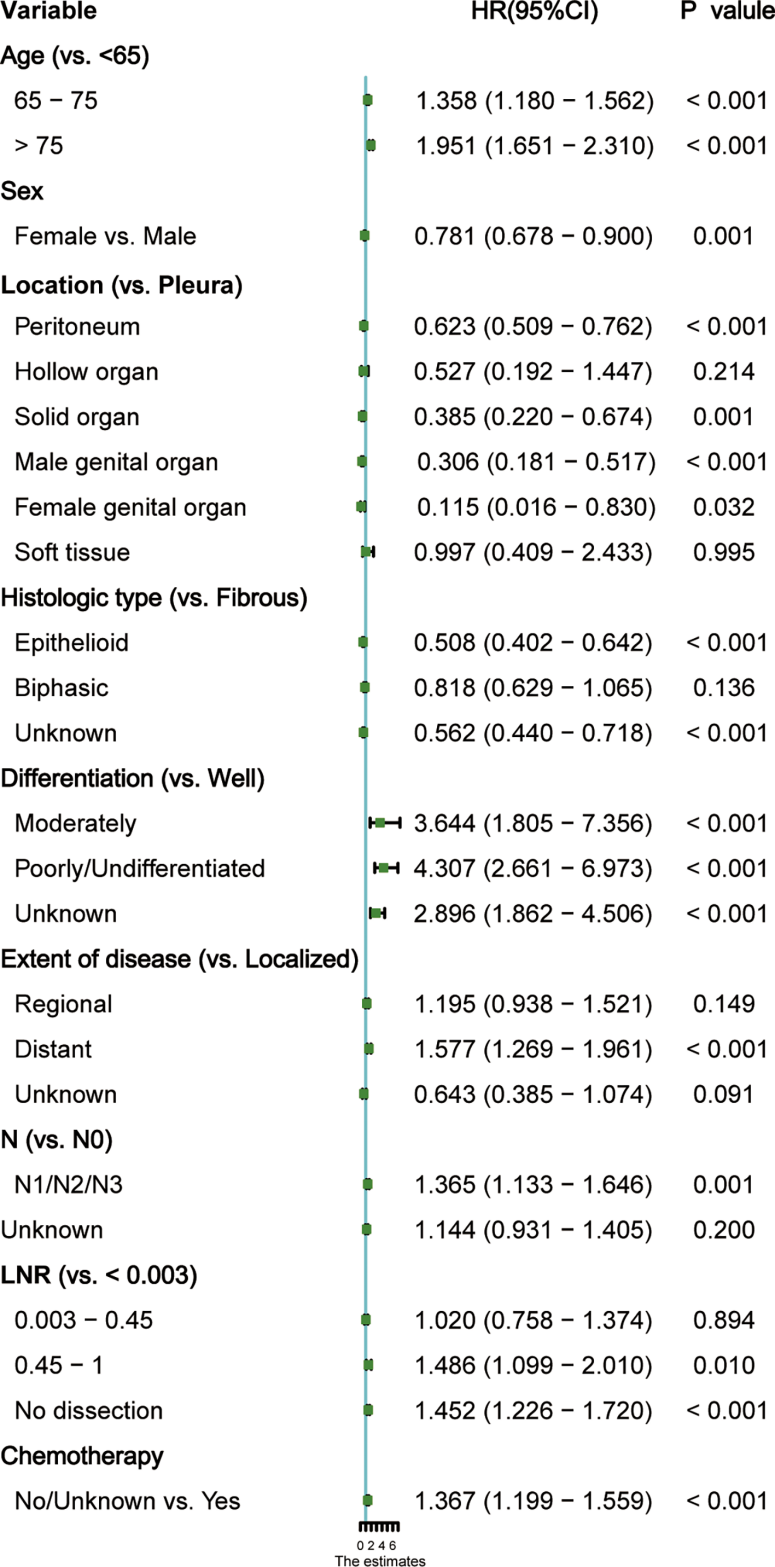
Forest plot of each factor’s ability to predict overall survival (OS) in multivariate Cox proportional hazards regression model. HR, hazard ratio; CI, confidence interval.

Several demographic and tumor characteristics were significantly associated with OS in the univariate Cox regression model (**Table 3**), including marital status (P = 0.02), age (P <0.001), gender (P <0.001), location (P < 0.001), histological type (P< 0.001), differentiation (P <0.001), extent of disease (P <0.001), stage (P <0.001), T (P <0.001), N (P <0.001), M (P <0.001), surgical type (P =0.007), lymph nodes dissection (P =0.002), number of positive nodes (P <0.001), LNR (P <0.001), and chemotherapy (P <0.001). The variables identified above were included in the stepwise Cox proportion regression, which showed that age, sex, location, histological type, differentiation grade, the extent of disease, N status, ratio, and chemotherapy were independent prognostic factors for OS (**Figure 3**).

**Table 3:** The ability of each variable to predict overall survival (OS) *via* the Univariate Cox proportional hazards regression analysis in the training cohort.

Variable	Univariate analysis		
	HR	95% CI	P
**Marital status**			0.02
Married	Ref	–	–
Divorced/Widowed/Separated	1.130	0.971 - 1.316	0.116
Unmarried	0.805	0.668 - 0.971	0.024
Unknow	0.903	0.649 - 1.256	0.544
**Race**			0.4
White	Ref	–	–
Black	0.845	0.651 - 1.097	0.206
Others	0.899	0.656 - 1.233	0.510
**Age, year**			< 0.001
<65	Ref	–	–
65 - 75	1.688	1.472 - 1.936	< 0.001
> 75	2.790	2.390 - 3.258	< 0.001
**Sex**			< 0.001
Male	Ref	–	–
Female	0.636	0.556 - 0.728	< 0.001
**Location**			< 0.001
Pleura	Ref	–	–
Peritoneum/Retroperitoneum	0.433	0.366 - 0.511	< 0.001
Hollow organ	0.476	0.178 - 1.271	0.139
Solid organ	0.478	0.282 - 0.811	0.006
Male genital organ	0.262	0.164 - 0.419	< 0.001
Female genital organ	0.056	0.008 - 0.395	0.004
Soft tissue	1.033	0.428 - 2.489	0.943
**Histologic type**			< 0.001
Fibrous	Ref	–	–
Epithelioid	0.385	0.309 - 0.480	< 0.001
Biphasic	0.683	0.529 - 0.882	0.004
Unknown	0.363	0.2878 - 0.459	< 0.001
**Differentiation**			< 0.001
Well	Ref	–	–
Moderately	5.449	2.725 - 10.900	< 0.001
Poorly/Undifferentiated	8.076	5.038 - 12.946	< 0.001
Unknown	5.022	3.256 - 7.747	< 0.001
**Extent of disease**			< 0.001
Localized	Ref	–	–
Regional	1.546	1.229 - 1.945	< 0.001
Distant	1.984	1.618 - 2.434	< 0.001
Unknown	0.770	0.467 - 1.270	0.306
**Stage**			< 0.001
I	Ref	–	–
II	1.197	0.954 - 1.502	0.120
III	1.013	0.837 - 1.228	0.892
IV	1.364	1.133 - 1.643	0.001
Unknown	0.632	0.507 - 0.788	< 0.001
**T**			< 0.001
T1	Ref	–	–
T2	1.105	0.920 - 1.329	0.285
T3	0.814	0.678 - 0.976	0.026
T4	1.355	1.121 - 1.637	0.002
Unknown	0.486	0.3945 - 0.598	< 0.001
**N**			< 0.001
N0	Ref	–	–
N1/N2/N3	1.374	1.197 - 1.578	< 0.001
Unknown	0.681	0.576 - 0.804	< 0.001
**M**			< 0.001
M0	Ref	–	–
M1	1.113	0.947 - 1.307	0.194
Unknown	0.505	0.415 - 0.615	< 0.001
**Surgical type**			0.007
Palliative	Ref	–	–
Radical	0.809	0.701 - 0.933	0.004
NOS	1.123	0.837 - 1.507	0.439
**Lymph nodes dissection**			0.002
Yes	Ref	–	–
No/Unknown	1.212	1.069 - 1.374	0.003
**Number of nodules dissected**			0.050
0	Ref	–	–
1 - 11	0.852	0.734 - 0.988	0.034
12- 81	0.867	0.725 - 1.038	0.120
**Number of positive nodules**			< 0.001
0	Ref	–	–
1 - 3	1.499	1.177 - 1.908	0.001
4 - 31	1.608	1.233 - 2.097	< 0.001
No dissection	1.407	1.199 - 1.650	< 0.001
**LNR**			< 0.001
<0.003	Ref	–	–
0.003 - 0.45	1.376	1.074 - 1.765	0.012
0.45 - 1	1.820	1.408 - 2.353	< 0.001
No dissection	1.410	1.202 - 1.653	< 0.001
**Radiation**			0.700
Yes	Ref	–	–
No/Unknown	0.727	0.834 - 1.135	0.727
**Chemotherapy**			< 0.001
Yes	Ref	–	–
No/Unknown	1.208	1.070 - 1.364	0.002

**Figure 4:**
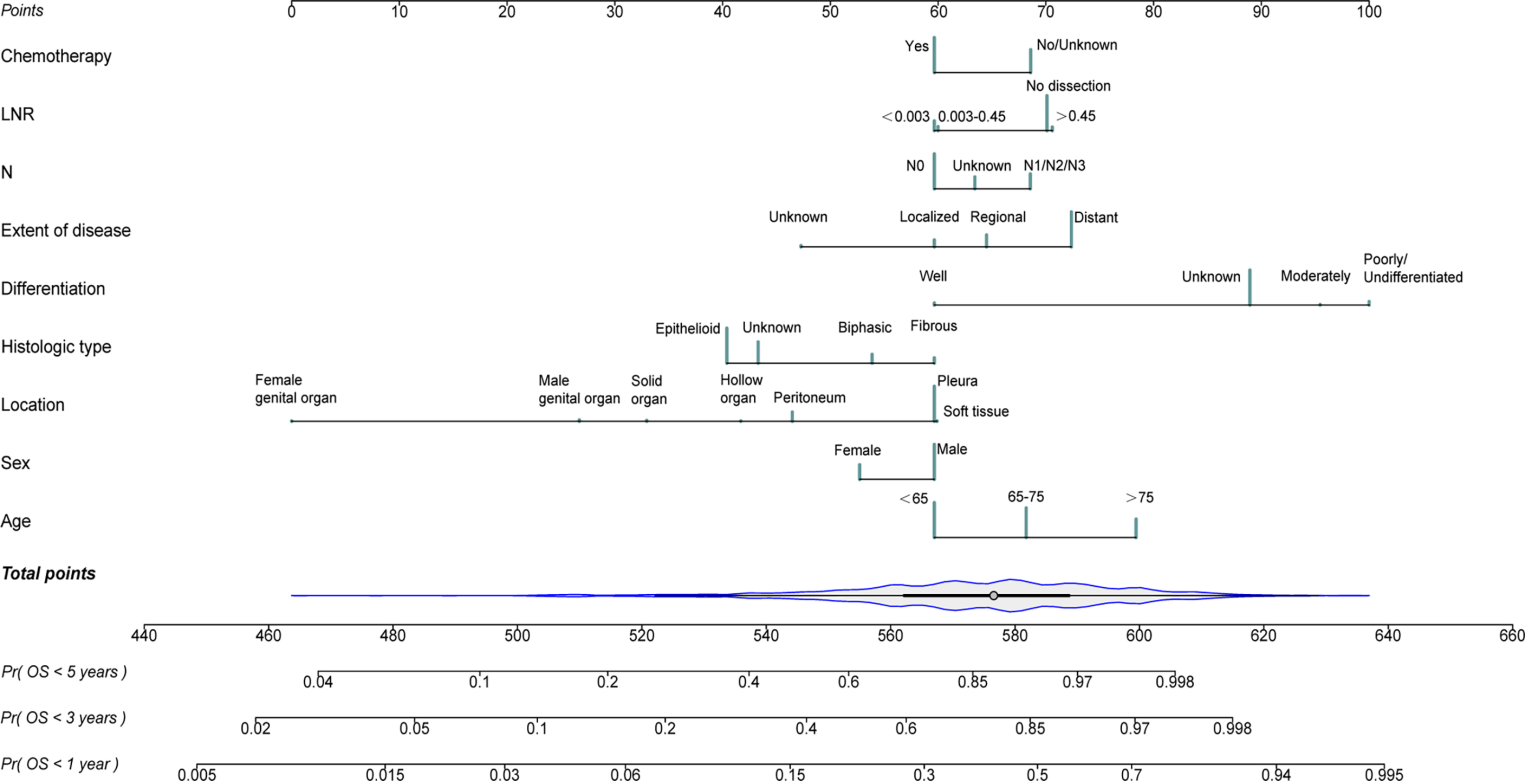
Nomogram for predicting 1-, 3- and 5- year probabilities of overall survival (OS) for patients with malignant mesothelioma (MM) originating from different primary sites.

The independent risk variables identified through the multivariate analysis were used to create a nomogram (**Figure 4**), which demonstrated that the extent of disease, differentiation, and location had a major impact on OS, while sex, N status, LNR, and chemotherapy only had a moderate effect. On the point scale, every variable subtype included was assigned a risk score. The overall points (ranging from 440 to 660) of patients were then determined. Finally, the 1-, 3-, and 5-year survival probability of individuals was predicted according to the total risk scores calculated.

### Model performance and validation

**Figure 5:**
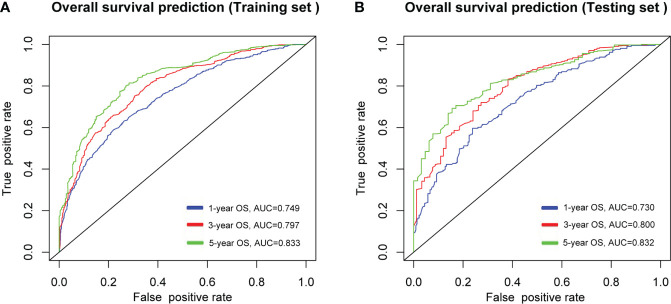
ROC curves of the nomogram in predicting the probability of overall survival (OS) at 1-, 3- and 5- year points in **(A)**, training cohort, and **(B)**, validation cohort. ROC, receiver operating characteristic curve; AUC, areas under the ROC curve.

As shown in **Figure 5**, the AUCs of the nomogram for 1-, 3-, and 5-year OS prediction in the development cohort were 0.749, 0.797, and 0.833, respectively, and the AUCs of the validation cohort were 0.730, 0.800 and 0.832 respectively. Additionally, C-indexes for the training and testing sets were 0.701 and 0.665, respectively. The nomogram featured good prediction accuracy for 1-, 3-, and 5-year OS probability in the training and testing cohorts.

**Figure 6:**
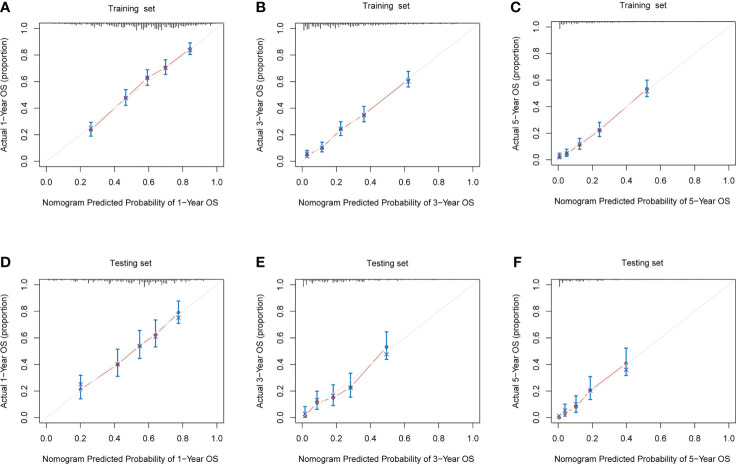
The calibration plots for prediction of 1-, 3- and 5- year overall survival (OS) probabilities between the nomogram and the actual observation in **(A–C)**, training cohort, and **(D–F)**, validation cohort.

Both in the training set (**Figures 6A–C**) and the testing set (**Figures 6D–F**), calibration plots of nomogram predicted probability of 1-, 3- and 5−Year OS illustrated an optimal consistency between the prediction and the observation.

## Discussion

MMe is a rare malignancy that mainly originates from the pleura but may also occur in other sites. So far, the majority of available prognostic nomograms are restricted to MMe arising in the pleura (7, 10). Our study has compared the OS of patients with MMe across multiple sites and found that the overall survival of malignant mesothelioma in different primary sites was different and found that patients with MMe occurring in soft tissues, hollow organs, or pleura have a worse prognosis than those originating from the peritoneum, solid organs, or genital organs.

The prognosis for MMe is dismal, and its diagnosis is notoriously difficult (3, 17). For example, the median survival time for malignant pleural mesothelioma is about 10-12 months (18), which is worse than reported in our study (**Table 2**). It may be because most (80%) patients with malignant pleural mesothelioma are typically diagnosed when the disease has progressed to an advanced stage (19). Individualized surgical treatment is not available for these patients (17, 20). Chemotherapy and other treatments also have a limited effect (21), thus leading to poor prognosis (19). The severe phase of MMe can be avoided by early identification of risk factors for the disease. Talha and Latif reported that early identification of asbestos exposure and its duration, erythrocyte sedimentation rate, and pleura-serum LDH ratio helps prevent patients from entering a high risk of MMe (22, 23). Based on co-expression patterns between ceRNA and immune cells, it was found that has-miR-582-5p, CASP9, dendritic cell rest, ANIX2, T cell CD8, and T cell CD4 memory rest may be associated with mesothelioma bone metastasis (24). Lai J also validated that alternative splicing (AS) events associated with the immune system can help clinicians diagnose MMe earlier and more accurately assess disease activity, and possibly even a potential therapeutic target for MMe (25). It is necessary to establish predictive models for the diagnosis, treatment or prognosis of malignant mesothelioma and its complications. Zhang X used four routinely detected variables, namely carcinoembryonic antigen (CEA) levels, monocyte counts, N-terminal parent-B natriuretic peptide (NT-pro-BNP) levels, and pleural effusion chloride levels at admission as predictors and developed the CONCH prognostic scoring system, which can guide the choice of malignant pleural effusion (MPE) interventions and management (26). Also, Talha ‘s research leverages the latest datasets from the Public Repository (UCI) to present a machine learning-based framework that eliminates the need for expensive testing and painful procedures and can help us make early diagnoses and better treat malignant mesothelioma through important prognostic factors (27). In our research, we developed the first predictive model for the survival outcome of MMe from different sites. This undoubtedly improved our understanding of MMe and provided a basis for the management and care of patients with MMe in different parts.

To identify the independent risk factors included in the variables of this study, the Cox proportional hazards model was employed. Our analysis found that old age, male, tumor originating from pleura, soft tissue or hollow organs, and LNR > 0.45 or no lymph node dissection were associated with shorter OS, whereas young age, female, localized disease, epithelial histological type, high differentiation, no lymph node metastasis and receiving chemotherapy favor a better prognosis (**Figure 3**).

A recent study described the differences in survival time between young and old malignant mesothelioma patients (28). Older MMe patients have a worse prognosis than younger patients (7, 10, 29). The multivariate analysis in our study yielded similar conclusions, with MMe patients older than 65 years having a relatively poor OS, whereas patients younger than 65 years had longer overall survival. Our findings corroborated a previous study identifying that the female gender was an independent factor associated with good survival outcomes (17, 30–33). This may be because estrogen in female patients in combination with the tumor suppressor estrogen receptor (ER)-β inhibits the progression of malignant mesothelioma (34, 35).

In this study, there was no inconsistency between clinical staging and actual pathological staging, as the pathological staging was obtained from patient information recorded in the Seer database (13, 14). Recent studies have shown that tumor volume in MMe may be closely related to patient staging, assessing treatment response, and predicting survival (36–38). Unfortunately, the present study did not validate this conclusion as tumor volume in MMe patients was not recorded in the SEER database. Some studies have shown that tumor TNM stage, size, and distant metastasis may be related to the survival time of MMe patients (10). However, no statistical significance was found between tumor TNM stage, T stage, and M stage and overall survival time of MMe patients in our study. Several previous studies have reported an association between positive lymph node pathology and reduced survival in MMe patients (39–41). Our study also found that MMe patients with lymph node stage N+ had a shorter survival time than patients without lymph node metastases. In addition, the prognosis of MMe patients was not significantly associated with the number of positive lymph nodes but was significantly associated with the ratio of the number of positive lymph nodes to the total number of detected lymph nodes. A multicenter study by Ilir Hysi et al. also demonstrated that overall survival was not related to the total number of lymph nodes involved and that a lower lymph node ratio (≤13%) implied a considerable improvement in median survival time. However, the lymph node ratio (LNR) was not an independent prognostic factor for patient survival outcomes. This may be attributed to only 99 patients being enrolled in their analysis and incomplete information such as recurrence, cohort heterogeneity, etc. (42).

Previous studies have reported that the histological type is an important factor affecting the survival time, and the prognosis of MMe patients with epithelial histological type is better than the non-epithelial type (43–47). Similarly, our study also found that the survival outcomes of MMe patients were closely related to their tissue type. Specifically, patients with epithelial histology had a better prognosis than those with fibroid and biphasic histology. Patients with epithelial histopathology have a better prognosis and are more responsive to treatment, but other types of mesothelioma patients should not be excluded from first-line trials (48). In addition, malignant mesothelioma (MMe) *in situ* may be a separate category (48, 49). Our study also found that the extent of diseases had a statistically significant difference, such as limited disease and regional MMe patients had a relatively good prognosis than those with distant metastases. Moreover, our study revealed the relationship between the prognosis of patients with MMe and the grade of tumor differentiation. A higher degree of tumor differentiation was associated with a better prognosis. Conversely, patients with poorly differentiated or undifferentiated tumors generally have shorter survival (7, 10, 44).

Some reports indicate that the incidence of MMe in some areas may have reached a peak. However, the long-term survival time of existing patients is still dismal, so the prognosis and quality of life of MMe patients cannot be ignored (31, 50, 51). And surgery-based multimodal therapy has a promising prognosis in selected patients with malignant mesothelioma (20, 52). All patients included in our study underwent surgery, but there was no significant difference in outcome between complete resection and palliative resection (53). It is worth mentioning that chemotherapy is an irreplaceable part of the current treatment of malignant mesothelioma. For patients who can undergo surgery, chemotherapy can further prolong their survival (32). For unresectable or advanced malignant mesothelioma, the current first-line chemotherapy regimen is pemetrexed plus cisplatin (54–56), and a complete chemotherapy regimen is associated with better survival outcomes (57). In contrast, Barsky et al. proposed a high BED (biologically effective dose) SBRT (stereotactic body radiation therapy) regimen for oligometastatic malignant pleural mesothelioma (58). Radiation therapy has also been shown to be useful after preoperative neoadjuvant therapy (20), extrapleural pneumonectomy (EPP) and less aggressive surgery (59). However, radiotherapy does not improve outcomes for patients with MMe (60). Our study also confirms that the prognosis of patients with malignant mesothelioma is closely related to chemotherapy, but not to radiotherapy. The first-line efficacy evaluation of ipilimumab plus nivolumab combination therapy showed promise for immune checkpoint inhibition and cell-based therapy (61). This was also confirmed by the recent approval of nivolumab plus ipilimumab combination therapy as a first-line treatment marker in patients with malignant pleural mesothelioma (55). A recent study evaluating CHECKMATE-743 found that the impact of this first-line therapy on quality of life and overall survival in patients with MPM remains controversial (62). Furthermore, tumor programmed death ligand 1 (PD-L1) expression is not predictive in clinical trials. This may be because MPM is a low tumor mutational burden (TMB) tumor (63). In addition, approximately 40% of patients treated with nivolumab plus ipilimumab experienced grade 3 or higher adverse events (AEs) (64).

## Limitations and future directions

Due to the low incidence of MMe, few studies have focused on the clinical characteristics and prognosis of MMe. However, the following limitations remain: (1) Our study is a retrospective analysis based on the Seer database, and selection bias is unavoidable. (2) The SEER database lacks detailed clinical data such as surgical methods, specific radiotherapy, and chemotherapy regimens, which may imply other unknown variables. (3) With advances in surgical (65–67) and treatment modalities (54, 61), patients with recently diagnosed MMe may have a better prognosis than those included in our study. (4) For the management of MMe patients, no normative standard has yet been established. There is no unified staging standard for MMe of different locations, which may avoid the overfitting of the model due to the inclusion of too many variables and affect the ease of use.

A large prospective trial with strict design needs to be carried out to verify our results. Information not recorded in the Seer database can be included in the trial, such as specific treatment plans, molecular pathology or blood indicators. Besides, external validation is needed to demonstrate its performance in clinical practice.

## Conclusion

In conclusion, this study is based on the Seer database, including large sample size, clear pathological diagnosis, and complete follow-up. Our study found that there were differences in prognosis for malignant mesothelioma at different anatomical sites. Further, age, sex, histological type, location, differentiation, the extent of disease, N status, ratio, and chemotherapy were identified as independent factors affecting the overall survival (OS) of MMe patients. Based on these factors, we built a nomogram that can be used to assess the prognosis of individual MMe patients at different sites. Prognostic model established in our study provides a reference for clinical decision-making and helps medical workers to individualize the care of patients with malignant mesothelioma.

## Data availability statement

The datasets presented in this study can be found in online repositories. The names of the repository/repositories and accession number(s) can be found in the article/supplementary material.

## Ethics statement

Ethical review and approval was not required for the study on human participants in accordance with the local legislation and institutional requirements. Written informed consent for participation was not required for this study in accordance with the national legislation and the institutional requirements.

## Author contributions

SS, YL and YW conceived and designed the experiments. SS, LS, KQ and TY collected and analyzed data. SS, YW and XJ wrote this manuscript. All authors contributed to the article and approved the submitted version.

## Funding

Funding from Outstanding Doctoral Project Funding Program of Air Force Military Medical University (2021D08) and Shandong Province Medical and Health Science and Technology Development Plan Project (2018WS383).

## Acknowledgments

The authors are grateful for the invaluable support and useful discussions with other members of the Department of Thoracic Surgery, and thank Home for Researchers (www.home-for-researchers.com) for language enhancement of this paper.

## Conflict of interest

The authors declare that the research was conducted in the absence of any commercial or financial relationships that could be construed as a potential conflict of interest.

## Publisher’s note

All claims expressed in this article are solely those of the authors and do not necessarily represent those of their affiliated organizations, or those of the publisher, the editors and the reviewers. Any product that may be evaluated in this article, or claim that may be made by its manufacturer, is not guaranteed or endorsed by the publisher.

## Abbreviations

MMe, malignant mesothelioma; OS, overall survival; SEER database, the Surveillance, Epidemiology, and End Results database; LNR, lymph node ratio; ROC, receiver operating characteristic curve; AUROC, the area under the ROC curve; BAP1, the BRCA1-associated protein 1; cN0, clinical N0 stage; MPM, malignant pleural mesothelioma; pN, pathological N stage; cN, clinical N stage; AIC, the Akaike information criterion; HRs, the hazard ratios; Cis, confidence intervals; AS, alternative splicing; CEA, carcinoembryonic antigen; NT-pro-BNP, N-terminal parent-B natriuretic peptide; MPE, malignant pleural effusion; ER, estrogen receptor; BED, biologically effective dose; SBRT, stereotactic body radiation therapy; EPP, extrapleural pneumonectomy; PD-L1, programmed death ligand 1; TMB, tumor mutational burden (TMB); AEs, adverse events.
